# The association between outdoor air pollution and lung cancer risk in seven eastern metropolises of China: Trends in 2006-2014 and sex differences

**DOI:** 10.3389/fonc.2022.939564

**Published:** 2022-09-29

**Authors:** Wei Wang, Liu Meng, Zheyu Hu, Xia Yuan, Weisi Zeng, Kunlun Li, Hanjia Luo, Min Tang, Xiao Zhou, Xiaoqiong Tian, Chenhui Luo, Yi He, Shuo Yang

**Affiliations:** ^1^ Gastroenterology and Urology Department II, Hunan Cancer Hospital/the Affiliated Cancer Hospital of Xiangya School of Medicine, Central South University, Changsha, China; ^2^ Gastroenterology and Urology Department II, Hunan Cancer Hospital/the Affiliated Cancer Hospital of Xiangya School of Medicine, Clinical Research Center For Gastrointestinal Cancer In Hunan Province, Changsha, China; ^3^ Scientifc Research Office, Hunan Cancer Hospital/The Affiliated Cancer Hospital of Xiangya Medical School, Central South University, Changsha, China

**Keywords:** outdoor air pollution, lung cancer, PM10, SO2 (sulphur dioxide), NO2

## Abstract

There is a positive association between air pollution and lung cancer burden. This study aims to identify and examine lung cancer risks and mortality burdens associated with air pollutants, including PM_10_, NO_2_ and SO_2_, in seven eastern metropolises of China. The study population comprised a population from seven eastern metropolises of China. The yearly average values (YAV, μg/m^3^) of the PM_10_, NO_2_ and SO_2_ levels were extracted from China Statistical Yearbook (CSYB) for each selected city from 2006 to 2014. Data collected in the China Cancer Registry Annual Report (CCRAR) provide lung cancer incidence and mortality information. A two-level normal random intercept regression model was adopted to analyze the association between the lung cancer rates and individual air pollutant concentration within a five-year moving window of past exposure. The yearly average values of PM10, SO2 and NO2 significantly decreased from 2006 to 2014. Consistently, the male age-adjusted incidence rate (MAIR) and male age-adjusted mortality rate (MAMR) decreased significantly from 2006 to 2014.Air pollutants have a lag effect on lung cancer incidence and mortality for 2-3 years. NO2 has the significant association with MAIR (RR=1.57, 95% CI: 1.19-2.05, p=0.002), MAMR (RR=1.70, 95% CI: 1.32-2.18, p=0.0002) and female age-adjusted mortality rate (FAMR) (RR=1.27, 95% CI: 1.08-1.49, p=0.003). Our findings suggested that air pollutants may be related to the occurrence and mortality of lung cancer. NO2 was significantly associated with the risk of lung cancer, followed by SO2. Air pollutants have the strongest lag effect on the incidence and mortality of lung cancer within 2-3 years.

## Background

Lung cancer is one of the most common cancers, causing nearly one in five cancer deaths and approximately 1.59 million deaths annually worldwide (1, 2) and China bears the heaviest burden of disease associated with lung cancer (3). China has the largest number of tracheal, bronchus, and lung (TBL) cancer deaths with 757,171 (887,752 – 638,741), accounting for 50% of the global TBL cancer deaths (3). Lung cancer is the result of a constellation of potential risk factors, including tobacco use, environmental factors and genetic predisposition (4, 5). Among these, smoking has been firmly established as the leading cause (2, 5). Accordingly, along with early-stage diagnosis and improved treatment, interventions have partly focused on the reduction of tobacco use, which has made great sense for preventing lung cancer (6). Additionally, promising approaches are identifying other modifiable determinants of lung cancer risks and a modifiable determinant of emerging interest is ambient air pollution (1), which was classified a group I carcinogen to humans by the International Agency for Research on Cancer (IARC) (7).

The high prevalence of air pollutants might be responsible for the increased incidence of lung cancer in the last decades (8). Urbanization and industrialization in China come at the cost of environmental pollution and air pollution is the major environmental hazard in urban areas. The distribution area of haze in China has reached 130000 square kilometers (9). Ambient air pollution represents a complex mixture of a broad range of carcinogenic and mutagenic substances (10). Pollution mix varies considerably from place to place, and people are exposed to different cocktails of pollutants across different cities (11). Among all air pollutants, the most commonly monitored are particulate matter (PM), sulfur dioxide (SO_2_) and nitrogen dioxide (NO_2_) (12).

A growing body of evidence indicates that ambient air pollutants pose a range of adverse effects on the mortality and morbidity of lung cancer (13–16). Several mechanisms have been suggested to explain the effect of air pollutants on cancer risk, such as chronic systemic inflammation, oxidative stress and DNA damage in tissues (10, 17–20). Results of several epidemiological studies have shown higher risks for lung cancer in association with various measures of air pollution (8, 21–23).

In a survey commissioned by Cancer Research, 35% of adults chose cancer as their most feared health problem, leading all other problems by a considerable margin (24). Thus, cancer as one of the major health conditions worsened by pollution exposure may hold particular sway with the public. Given the ubiquity of air pollution exposure and the enormous public health burden of lung cancer, we conducted a population-based retrospective study in seven cities in China, which differ in terms of pollutant concentrations, to assess whether air pollution exposure is associated with incident lung cancer and lung cancer-related mortality. The study aims to identify and examine lung cancer risks and mortality burdens associated with air pollutants including PM_10_, NO_2_ and SO_2_, in China. The association between lung cancer burden and air pollution may shift public perceptions and ultimately help to promote policy development on air quality.

## Methods

### Air pollution data sources

We conducted the analysis using the database from China Statistical Yearbook (CSYB, National Bureau of Statistics) and the National Department of Environment (NDE). The annual concentrations of PM_10_, NO_2_ and SO_2_ were reported at the regulatory air pollution monitoring sites. In this study, annual air pollutants data from seven cities were retrospectively collected for consecutive nine years from 2006 to 2014. According to the relevant regulations of the Chinese government, the locations of the air pollutant monitoring stations were far away from traffic and industrial pollution sources. Therefore, their records were not affected by the local pollution sources, buildings, large-scale emissions such as coal, boilers or incineration plants. The monitoring data of these stations reflected the average level of the urban air pollution in China. There was no missing data for the selected seven cities from 2006 to 2014.

### Study population

The study population comprised urban residents in seven cities, respectively located in the Beijing-Tianjing-Hebei region (Beijing), the Yangtze River Delta region (Shanghai and Hangzhou), the Pearl River Delta region (Guangzhou), the Northeast region (Shenyang and Harbin) and the Central eastern region (Wuhan). These cities were chosen because they represented the Northeast Plain, the North Plain, the middle and lower reaches of the Yangtze River Plain, and the Pearl River delta plain. Most of them are located in the coastal areas of China, with the same or similar longitudes. They are all located in the plain areas with similar altitudes, representing different haze areas in the north and south of China. More importantly, there are approximately 100 million people living and working in these seven cities, which are highly representative of Chinese metropolises’ population.

### Air pollutant variables

The yearly average values (YAV, μg/m^3^) of PM_10_, NO_2_ and SO_2_ levels from 2006 to 2014 were extracted from CSYB. Since the tumorigenesis process always takes several years, in this study a five-year moving window of past exposure was taken into consideration for each pollutant. The YAVs of PM_10_, NO_2_ and SO_2_ in the 5 years preceding the outcome assessment were candidate variables for evaluating the effect of air pollutants on the lung cancer incidence and lung cancer-related mortality.

### Outcome measurements

Incidence (mortality) just measures new cases (deaths) of a disease that develop over a period of time without considering the denominator population. Incidence (mortality) rate is a measure of how quickly cases (deaths) of a disease of interest occurs, which reflects the speed of transition from disease-free (alive) to affected state (dead). We obtained the information of annual lung cancer incidence rate and mortality rate in selected cities from China Cancer Registry Annual Report (CCRAR) published by the National Cancer Registry Center (25–33). The annual lung cancer-related statistics included the crude incidence rate (CIR), crude mortality rate (CMR), male age-adjusted incidence rate (MAIR), female age-adjusted incidence rate (FAIR), male age-adjusted mortality rate (MAMR) and female age-adjusted mortality rate (FAMR). The detailed data collection and quality evaluation methods were referenced in the annual reports. There is no missing data for the selected cities from 2006 to 2014.

### Statistical analysis

All the selected cities except the northeastern region are at the top of China of economic development and have developed their economies mainly through trade, service or technology rather than industry. Besides, people who live in industrialized cities and cosmopolitan cities like Shanghai receive different air pollution levels, types and compositions, so the differences must be considered. In this study, a two-level random intercept regression model was developed to figure out the overall trend, the difference between cities, and explain the variability in the air pollution trend among cities.

A two-level normal random intercept regression model was adopted to analyze the association between pollutant concentrations within the 5 years preceding the outcome assessment and annual lung cancer-related statistics, including CIR, CMR, MAIR, MAMR, FAIR and FAMR. A random intercept model is also known as a two-level variance component model: y_ij_=μ+θ_i_+βx_ij_+ϵ_ij_,θ_i_~N(0,τ^2^), ϵ_ij_~N(0,σ^2^), where μ represents the overall intercept (grand mean), θ_i_ indicates the difference between the mean of city i and the grand mean, β is the vector of coefficients that do not vary between groups, x_ij_ indicates the fixed variables (e.g, 5-year prior NO_2_). Here, the estimate of β represents the effect of air pollutions (x_ij_), which is calculated by the random intercept model (different cities are supposed to have different intercept with a mean of μ and a variance of θ_i_. ϵ_ij_ represents the residual error, τ^2^ is the heterogeneity variance that represented the between-city variability in the intercept, and σ^2^ is the residual variance that represented the within-city variability in the residuals. The intraclass correlation ρ=τ2(τ2+σ2) measured the degree of similarity among same-city observations compared to the residual error σ^2^. The heterogeneity τ^2^ controlled the amount of shrinkage and how much information to borrow across cities. If the between-city variance τ^2^ was not considered, the standard error would be inflated and the p-value became too large. Therefore, compared to fixed estimates, the random effects estimators were more precise and minimize the total mean-square error (MSE). Statistical analyses were conducted by using R3.3.2 software. All tests of hypotheses were two-tailed and conducted at a significance level of 0.05.

## Results

According to CCRAR, the trend of the crude lung cancer incidence rate (CIR) and crude mortality rate (CMR) increased significantly from 2006 to 2014 (CIR: RR = 7.46, 95% CI: 3.82-14.59, p<0.0001; CMR: RR = 4.31, 95% CI: 2.59-7.10, p<0.0001; **Table 1**). However, when stratified by gender and adjusted for age, the female age-adjusted mortality rate (FAMR) and female age-adjusted incidence rate (FAIR) had no significant trend from 2006 to 2014, while the male age-adjusted incidence rate (MAIR) and male age-adjusted mortality rate (MAMR) decreased significantly from 2006 to 2014 (MAIR: RR = 0.50, 95% CI: 0.30-0.84, p=0.01; MAMR: RR =0.51, 95% CI: 0.32-0.81, p=0.006, respectively, **Table 1**).

**Table 1:** The integrated year trend of lung cancer risks in seven Chinese cities.

Measurements	Year trend (2006-2014)	
	RR (95% CI)	P value
**CIR**	7.46 (3.82, 14.59)	<0.0001
**CMR**	4.31 (2.59, 7.10)	<0.0001
**MAIR**	0.50 (0.30, 0.84)	0.01
**MAMR**	0.51 (0.32, 0.81)	0.006
**FAIR**	0.91 (0.68, 1.23)	0.55
**FAMR**	0.77 (0.59, 1.01)	0.06

RR represents rate ratio: the ratio of the incidence and mortality rate at one-unit increase of numeric variable versus the incidence and mortality rate at baseline. P value was calculated by using the two-level random intercept regression analysis. CIR, crude incidence rate; CMR, crude mortality rate; MAIR, male age-adjusted incidence rate; FAIR, female age-adjusted incidence rate; MAMR, male age-adjusted mortality rate; FAMR, female age-adjusted mortality rate.

**Figure 1:**
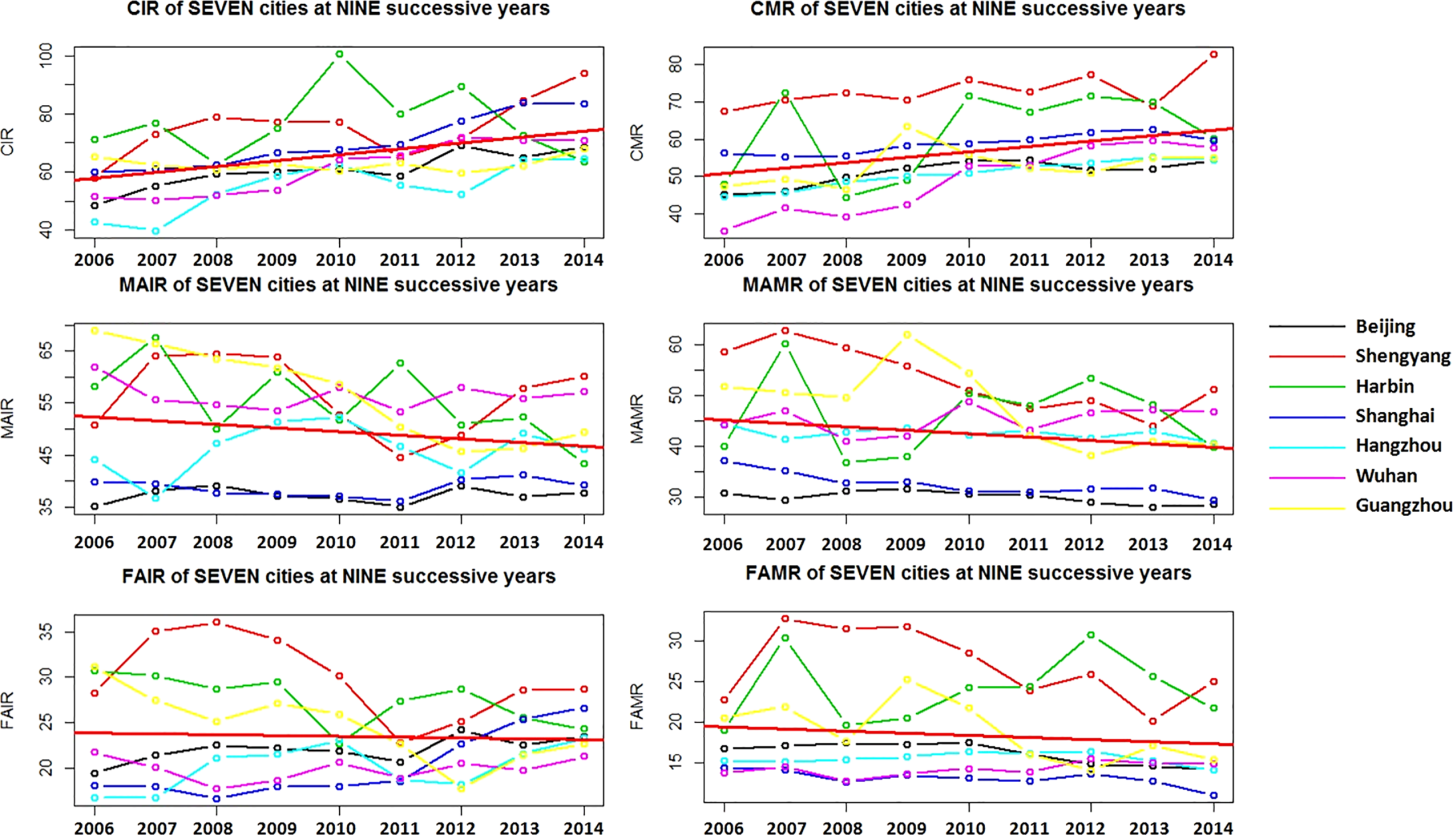
Year trends of lung cancer incidence and mortality in the seven selected cities. The Y-axis (%) was fitted with data range. The red bold line represented the estimated year trend based on integrated regression of seven cities.

**Figure 1** shows the illustrative yearly curves for the city specific-CIRs, CMRs, MAIRs, MAMRs, FAIRs and FAMRs from 2006 to 2014. As for the overall trends of the seven cities (red solid line), the CIR and CMR had an increased trend, while the MAIR and MAMR had a decreased trend. From 2006 to 2014, industrial cities such as Shenyang (red dash) and Harbin (green dash) had higher values for the CIR, CMR, MAIR, MAMR, FAIR and FAMR. In particular, Shenyang had the highest risks of lung cancer. Beijing and Shanghai had the lowest MAIR and MAMR. Wuhan had the lowest FAIR, and Shanghai had the lowest FAMR. In 2014, both the CIR and CMR in Beijing, Hangzhou and Guangzhou were lower than those in Shenyang. These findings suggested that the urban dwellers in Shenyang might have a higher risk of lung cancer than those in other large nonindustrial cities.

**Figure 2:**
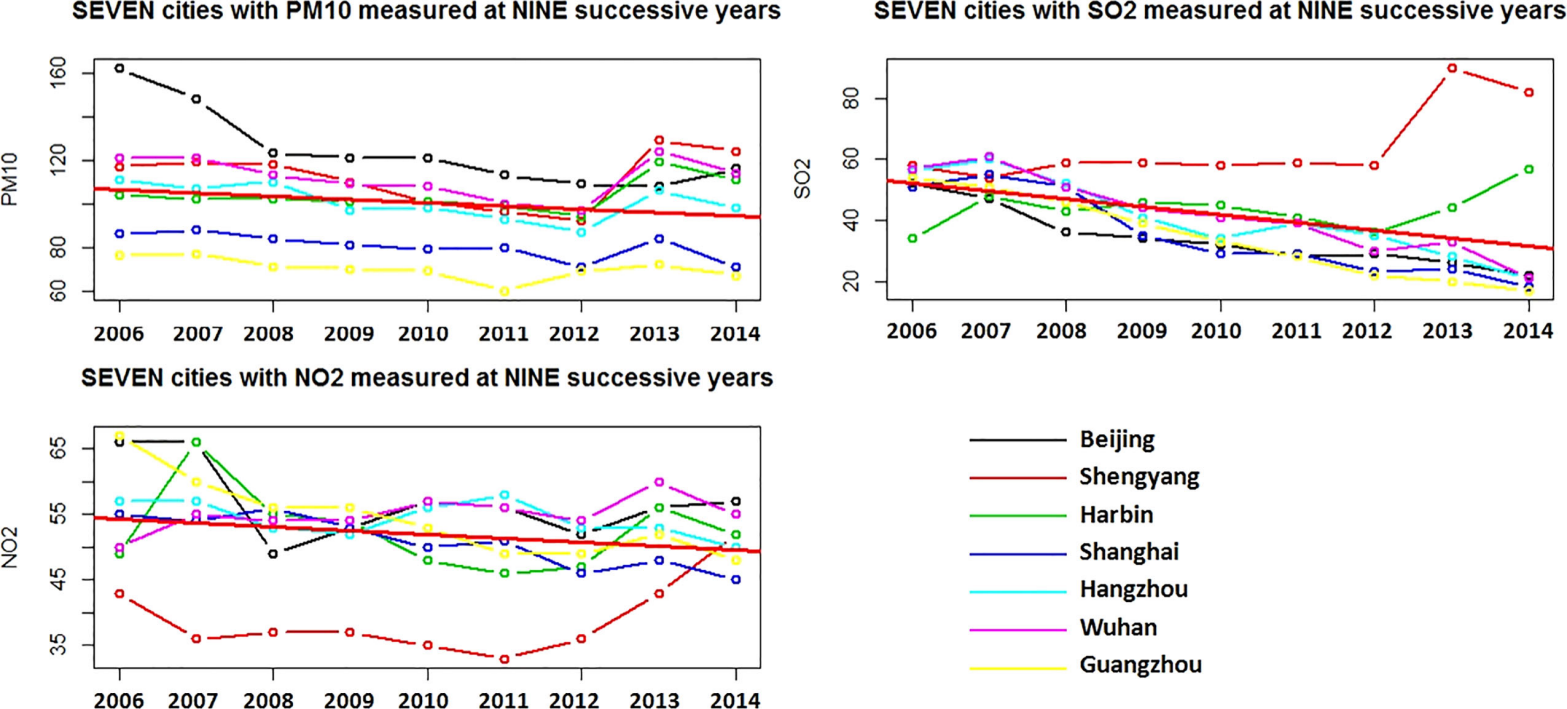
Year trends of air pollutants PM_10_, SO_2_ and NO_2_ in seven selected cities. The Y-axis (%) was fitted with data range. The red bold line represented the estimated year trend based on integrated regression of seven cities.

The yearly trends in all these air pollutants significantly decreased from 2006 to 2014 (PM_10_: estimated coefficient = -1.47, 95% CI: -0.53,-2.40, p=0.003; SO_2_: estimated coefficient = -2.56, 95%CI: -3.51, -1.61, p<0.0001; NO_2_: estimated coefficient = -0.59, 95% CI: -1.04, -0.13, p=0.015; **Table 2**). As shown in **Figure 2**, the air pollutants PM_10_, SO_2_ and NO_2_ had a decreasing trend from 2006 to 2014, while there was a peak in 2013, especially for SO_2_ and NO_2_ in Shenyang. The annual trends of the air pollutants were consistent with the annual trend of MAIR, MAMR and FAMR, but not for CIR and CMR, suggesting a potential correlation of the air pollutants with lung cancer-related MAIR, MAMR and FAMR.

**Table 2:** The integrated year trend of PM_10_, SO_2_ and NO_2_ in seven Chinese cities.

Measurements	Year trend (2006-2014)	
	β coefficients (95% CI)	P value
**PM_10_**	-1.47 (-0.53, -2.40)	0.003
**SO_2_**	-2.56 (-3.51, -1.61)	<0.0001
**NO_2_**	-0.59 (-1.04, -0.13)	0.015

β coefficients of the random intercept model [y_ij_=μ+θ_i_+βx_ij_+ϵ_ij_,θ_i_~N(0,τ^2^), ϵ_ij_~N(0,σ^2^)] represented the yearly trend (x_ij_) of pollutants (y_ij_). Negative β coefficient suggested a decreasing year trend.

**Figure 3:**
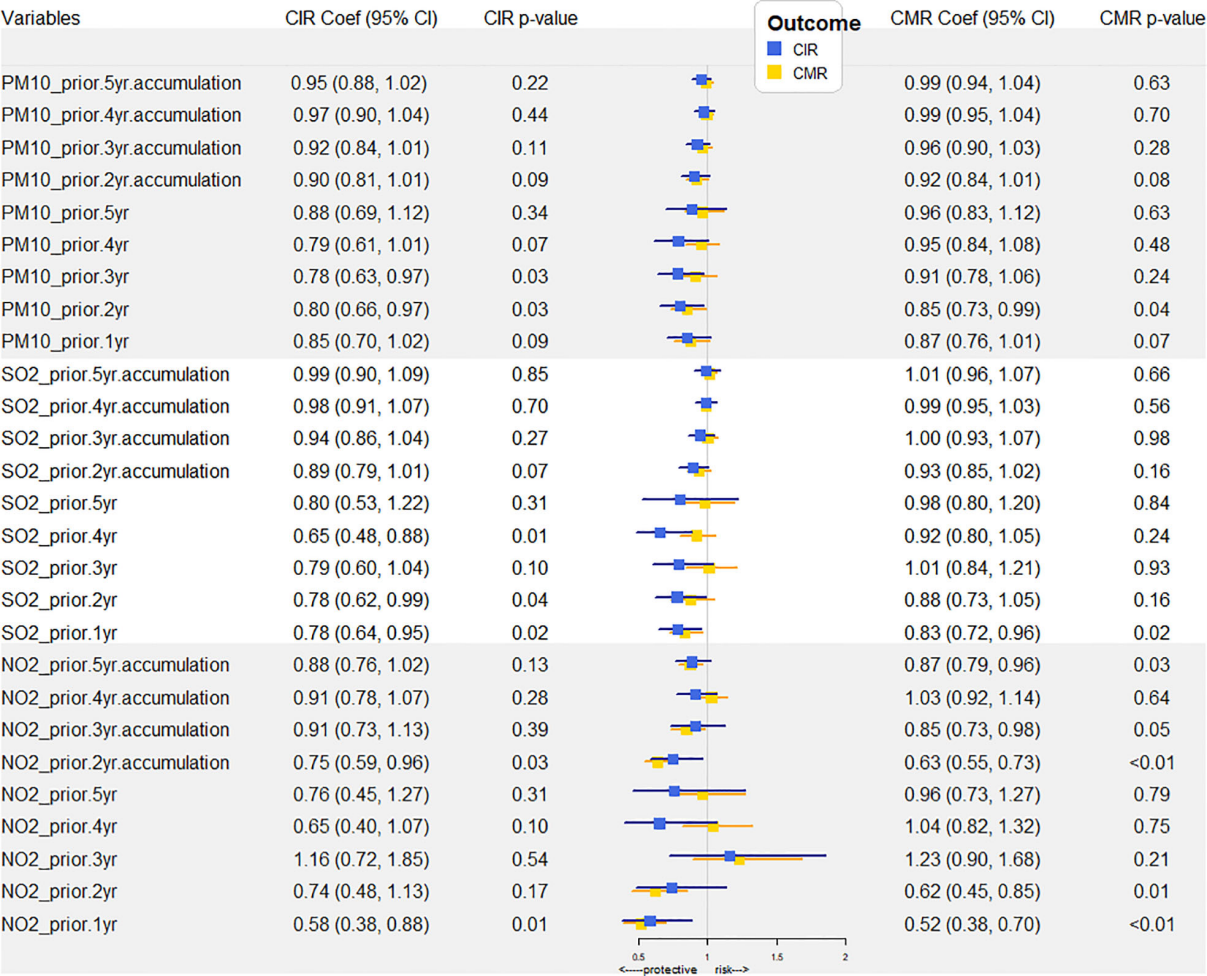
Air pollutant exposure-related RRs of CIR and CMR with a 5-year moving window. YAV, yearly average values (μg/m^3^); RR, risk ratio (with 95% CI). CIR, crude incidence rate; CMR, crude mortality rate.

We further investigated the potential effects of the air pollutants within a 5-year moving window of past exposure on lung cancer. The forest plot of the association between the concentrations of PM_10_, SO_2_, NO_2_ 0-5 year (s) before and CIR, CMR of lung cancer was shown in **Figure 3**. As shown in **Supplementary Table 1**, SO_2_ exposure 4 years before, 2 years before and 1 year before and during the present year, PM_10_ exposure 3 years before and 2 years before, and NO_2_ exposure 1 year before seemed to be protective factors for the lung cancer CIR. PM_10_ exposure 2 years before, NO_2_ exposure 1 year and 2 years before, SO_2_ exposure 1 year before and during the present year seemed to be protective factors for the lung cancer CMR. However, from common sense, air pollutants should be harmful to health. Because the lung cancer burden was heavy among the aging population, thus it would be not practicable to delineate the relationship between air pollution and lung cancer incidence and mortality without age-adjustment. Moreover, the time-trends of air pollutants and the age-adjusted statistics for MAIR, MAMR, FAIR and FAMR consistently decreased from 2006 to 2014, suggesting a positive correlation between air pollution and cancer burden.

**Figure 4:**
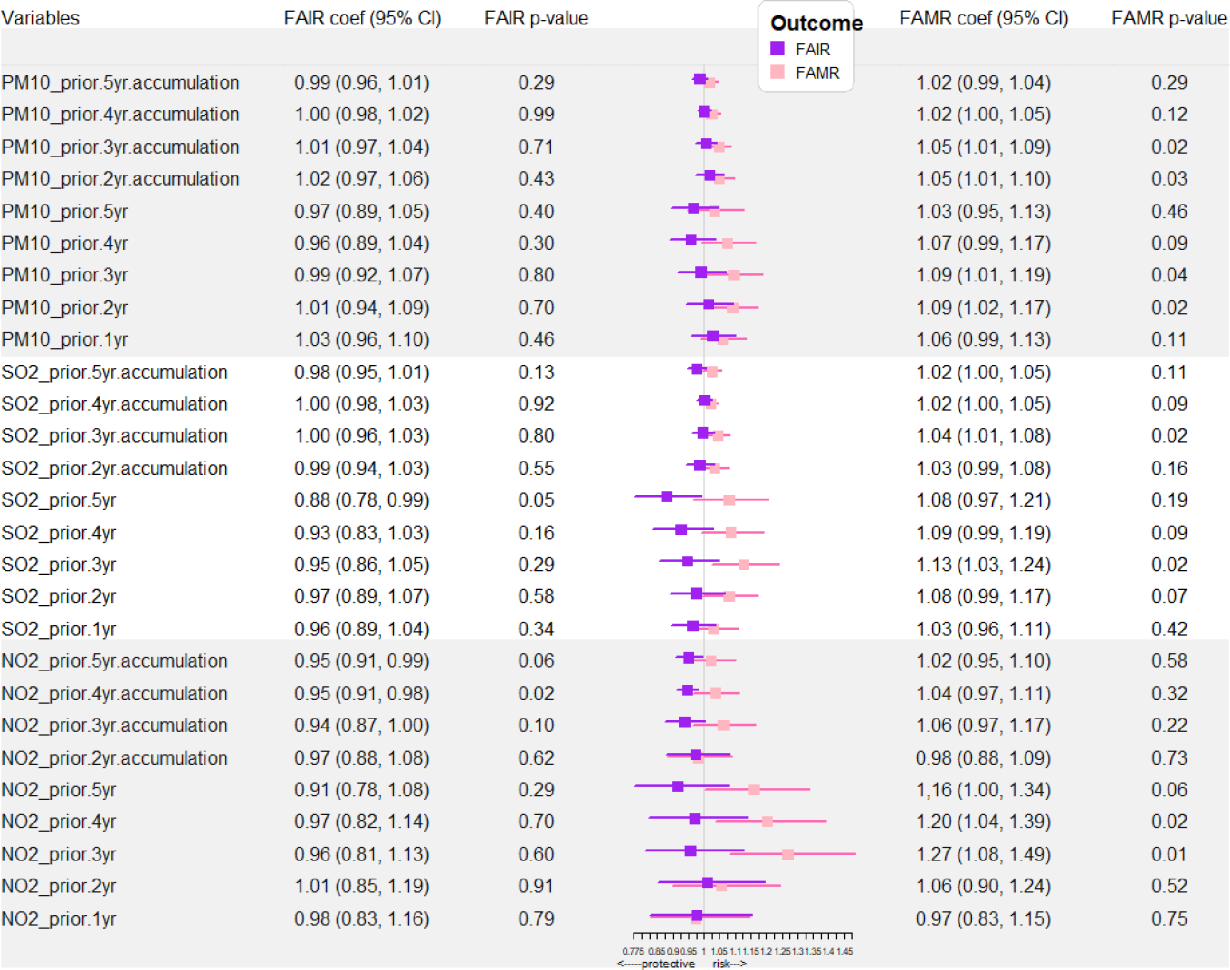
Air pollutant exposure-related RRs of FAIR and FAMR with a 5-year moving window. YAV, yearly average values (μg/m^3^). RR, risk ratio (with 95% CI); FAIR, female age-adjusted incidence rate; FAMR, female age-adjusted mortality rate.

The forest plot of the association between the concentrations of PM_10_, SO_2_, NO_2_ 0-5 year (s) before and FAIR, FAMR of lung cancer was shown in **Figure 4**. **Supplementary Table 2** demonstrated the significant risky air pollution for FAMR. The significant risky air pollution included: NO_2_ 5 years before (RR = 1.16, 95% CI: 1.00-1.34, p=0.05), NO_2_ 4 years before (RR = 1.20, 95% CI: 1.04-1.39, p=0.02), NO_2_ 3 years before (RR = 1.27, 95% CI: 1.08-1.49, p=0.01), PM_10_ 3 years before (RR = 1.09, 95% CI: 1.01-1.19, p=0.04), PM_10_ 2 years before (RR = 1.09, 95% CI: 1.02-1.17, p=0.02), and SO_2_ 3 years before (RR = 1.13, 95% CI: 1.03-1.24, p=0.02). Based on these analyses, we found that the YAV of air pollutants several years before the outcome assessment might be a significant risk factor for male and female age-adjusted lung cancer-related mortality.

**Figure 5:**
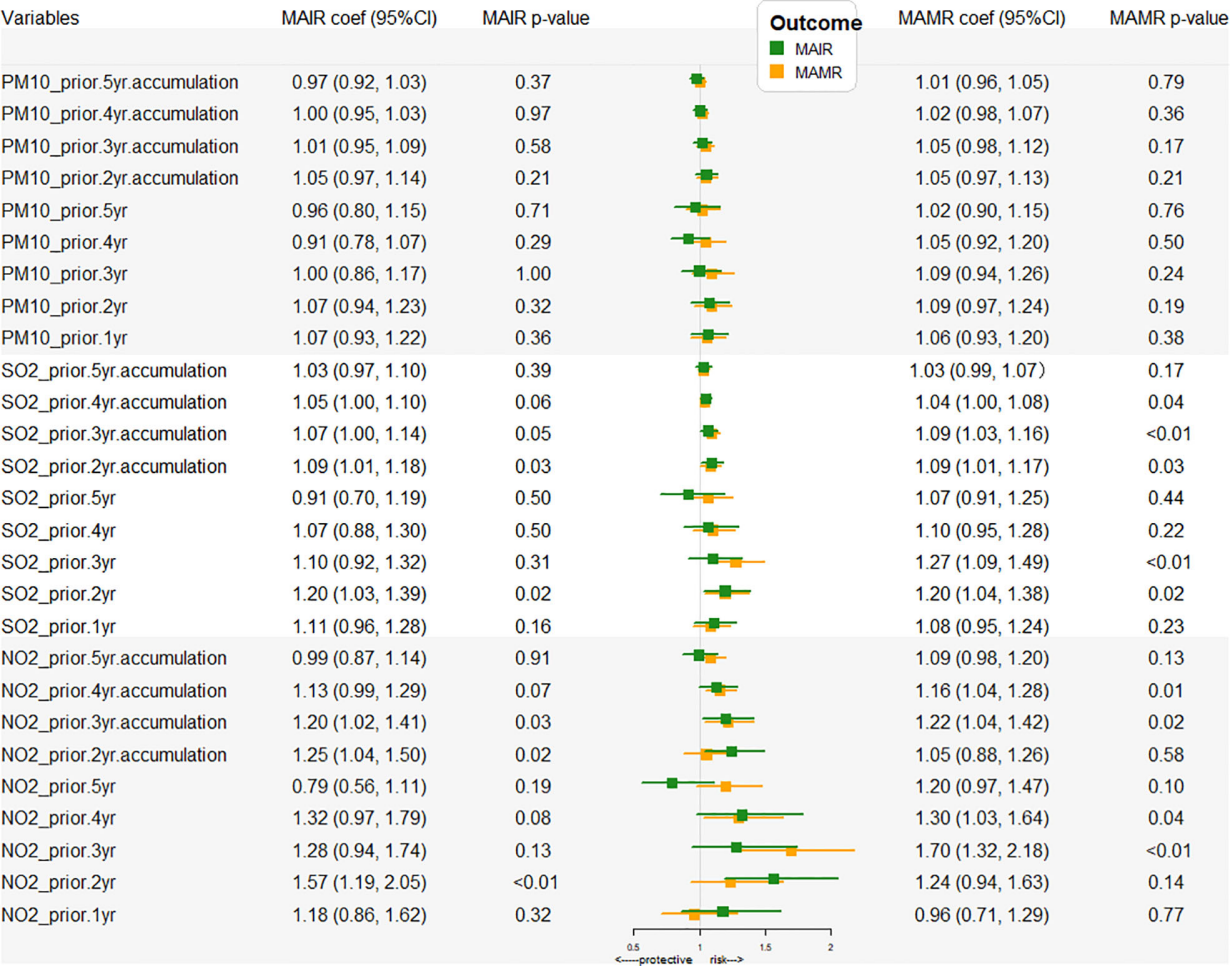
Air pollutant exposure-related RRs of MAIR and MAMR with a 5-year moving window. YAV, yearly average values (μg/m^3^). RR, risk ratio (with 95% CI); MAIR, male age-adjusted incidence rate; MAMR, male age-adjusted mortality rate.

The forest plot of the association between the concentrations of PM_10_, SO_2_, NO_2_ 0-5 year (s) before and MAIR, MAMR of lung cancer was shown in **Figure 5**. As shown in **Supplementary Table 3**, the risky air pollutions were listed. The significant air pollution risk factors for MAIR included SO_2_ 2 years before (RR = 1.20, 95% CI: 1.03-1.39, p=0.02), NO_2_ 2 years before (RR = 1.57, 95% CI: 1.32-2.05, p=0.002), and NO_2_ during the present year (RR=1.38, 95% CI: 1.04-1.83, p=0.03). The significant air pollution risk factors for MAMR included NO_2_ 4 years before (RR = 1.30, 95% CI: 1.03-1.64, p=0.04), NO_2_ 3 years before (RR = 1.70, 95% CI: 1.32-2.18, p=0.0002), SO_2_ 3 years before (RR = 1.27, 95% CI: 1.09-1.49, p=0.004), and SO_2_ 2 years before (RR = 1.20, 95% CI: 1.04-1.38, p=0.02). In terms of the age-adjusted lung cancer incidence rate, the NO_2_ and SO_2_ within the 2-year exposure window were significant risk factor for males not females. These findings suggested that high NO_2_ and SO_2_ exposure within 2 years is related to lung cancer occurrence, and high NO_2_ and SO_2_ exposure within three years also increased lung cancer-related mortality; however, PM_10_ was not significant.

**Figure 6:**
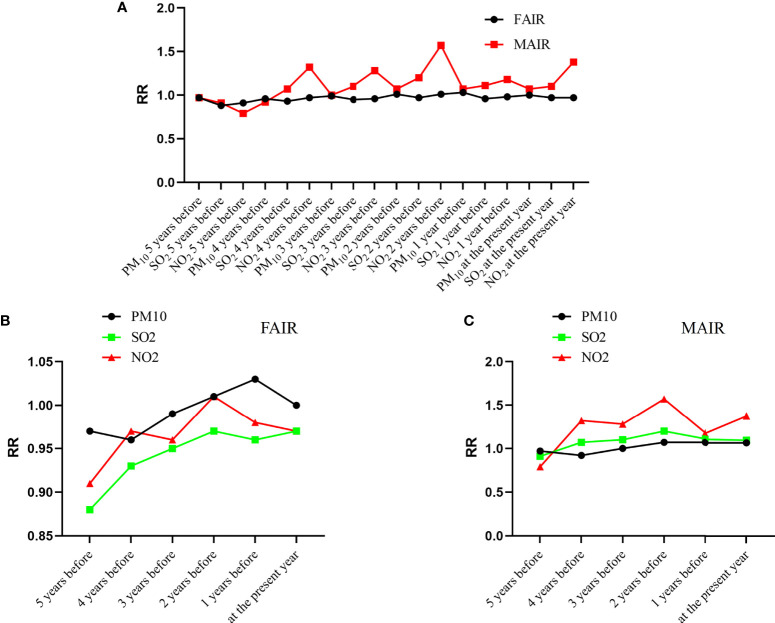
Air pollutant-related lung cancer incidence within a 5-year moving exposure window. **(A)** RR values of FAIR and MAIR related to the air pollutants; **(B)** RR values of FAIR related to individual air pollutants PM_10_, SO_2_ and NO_2_; **(C)** RR values of MAIR related to individual air pollutants PM_10_, SO_2_ and NO_2_. RR, risk ratio; FAIR, female age-adjusted incidence rate; MAIR, male age-adjusted incidence rate.

To further discriminate the impact of individual air pollutant on the sex- and age-adjusted lung cancer incidence and mortality, the RRs of MAIR, MAMR, FAIR and FAMR were individually presented in curves within a 5-year moving window of past exposure. As shown in **Figure 6A**, MAIR had higher RRs in the 5-year moving window, indicating stronger effects of air pollutants on MAIR than on FAIR. As shown in **Figures 6B, C**, the highest RR for both MAIR and FAIR came at the yearly concentration of air pollutant two years before, indicating the most significant lag effect for two years on lung cancer incidence. As shown in **Figure 6C**, NO_2_ exposure had the highest RR value for both MAIR.

**Figure 7:**
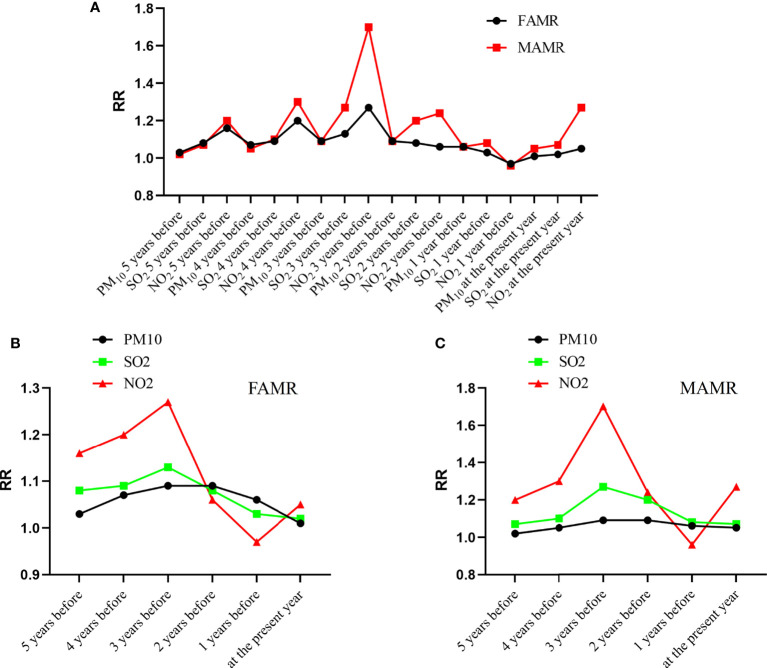
Air pollutant-related lung cancer mortality within a 5-year moving exposure window. **(A)** RR values of FAMR and MAMR related to the air pollutants; **(B)** RR values of FAMR related to individual air pollutants PM_10_, SO_2_ and NO_2_; **(C)** RR values of MAMR related to individual air pollutants PM_10_, SO_2_ and NO_2_. RR, risk ratio; FAMR, female age-adjusted mortality rate; MAMR, male age-adjusted mortality rate.

As shown in **Figure 7A**, MAMR had higher RRs in the 5-year moving window, indicating stronger effects of air pollutants on MAMR than on FAMR. As shown in **Figures 7B, C**, NO_2_ exposure had the highest RR value for both MAMR and FAMR, indicating that the NO_2_ level was the most important air pollutants affecting lung cancer-related mortality. In **Figures 7B, C**, the time effect of environmental pollutants on lung cancer mortality rate presents an inverted “U” structure. The highest RR for both MAMR and FAMR came at the yearly concentration of air pollutant 3 years before, indicating that the effects of air pollutants on MAMR and FAMR presented time lag distribution with the most significant lag effect for three years.

## Discussion

According to the annual report data from the China Statistical Yearbook, the official Chinese government air quality testing stations obligatorily report, the air pollutants PM_10_, SO_2_ and NO_2_ had overall decreasing trends year by year in seven major cities in China from 2006 to 2014. These trends were closely related to the great efforts made by the state and the general public in regard to environmental sanitation in recent years.

Air pollutants have been identified as group I carcinogens for lung cancer by International Agency for Research on Cancer (IARC) (24). According to the annual report of China Cancer Registry (CCRAR), we are surprised to find that crude lung cancer incidence and mortality rate are increasing year by year in these seven cities and are not in line with the concentrations of PM_10_, SO_2_ and NO_2_.

This is probably attributable to the aging population structure. Aging is a firmly established risk factor for cancers, and aging population have higher absolute incidence rates of cancer (34). The population age structure is introduced as an important control variable for cancer risks. China has been in the ranks of aging society and has a large aging population, even at an increasing speed of aging (35). Thus, it is advisable to adopt age-adjusted cancer incidence and mortality rates to evaluate the effects of cancer risk factors, as suggested in previous studies (36, 37). Therefore, the incidence and mortality rates of lung cancer were modified by age according to a standard world population structure.

The age-adjusted incidence rate and mortality rate of male lung cancer decreased year by year in the past ten years, consistent with the trend of air pollution concentration, suggesting that air pollutants may be related to the occurrence and mortality of male lung cancer. Although air pollutants had an overall decreasing trend in the seven major cities, the air pollution indicators of several industrial cities had peak levels from 2013 to 2014, especially in the northern industrial city such as Shenyang. From 2013 to 2014, the concentrations of SO_2_ and NO_2_ in Shenyang were significantly increased. The annual lung cancer incidence rate and mortality rate rose consistently during this period. The age standardized incidence rate and survival rate are common international measures for studying malignant tumors (38). In fact, they are more practicable in the correlation analysis between air pollutants and lung cancer.

Gender is another important control variable for cancer risk. The time-trends of air pollutants and the age-adjusted statistics MAIR/MAMR consistently decreased from 2006 to 2014. The female age-adjusted mortality rate (FAMR) and female age-adjusted incidence rate (FAIR) had no significant trend from 2006 to 2014. Additionally, we found that YAV of SO_2_ and NO_2_ at 2-years before the outcome were most associated with lung cancer incidence in males without significant effects on lung cancer incidence in females. The findings of our study suggested that compared to women, men may have a higher risk of lung cancer following exposure to ambient air pollution. YAV of SO_2_ and NO_2_ at 3-years before the outcome had the most significant effects on lung cancer-related mortality in males and females, but air pollutants presented stronger effects on males than females. Our results showed males may be more sensitive to air pollutants than females.

There are gender differences in the pathogenesis of smoking-related lung cancer. Female smokers suffer a higher risk of lung cancer than male smokers. There is insufficient evidence of gender differences in air pollutant-related lung cancer risks. Until now, sex-specific observations were not consistent among study locations and health outcomes. Some studies reported a larger mortality effect in males because of higher exposure associated with more outdoor activities, but some other studies also observed a stronger effect among females (39, 40). Our finding is consistent with most previous studies that reported slightly stronger health effects of air pollution for males compared to females (41). However, inconsistent results were observed in single-city studies in Shanghai, Guangzhou and Beijing in China (42–44).

The gender differences might be due to biological (e.g., physiopathological responses), demographic and behavioral differences (e.g., type of occupations, smoking, and lifestyle) between males and females (42, 45). Gender differences may be partly attributed to physiological differences (46). For instance, estrogens protect against the development of lung cancer (47). On the other hand, all major known risk factors, including smoking and occupational exposures, are more prevalent in males. The underlying reasons are still unclear and need to be further investigated.

The effects of single air pollutants are difficult to disentangle in an epidemiological study because pollutants are part of complex mixtures. The pollution mix varies considerably from place to place-from Shenyang to Beijing to Shanghai to Hangzhou to Wuhan, people are exposed to different cocktails of pollutants across different cities. PM_10_, NO_2_ and SO_2_ represent different characteristics of the air pollution mixture, which may be related to the source of the pollution variability. In our study, it seems likely that NO_2_ is the most important component for lung cancer risk and is more consistently associated with mortality than PM_10_ and SO_2_. In the correlation analysis between air pollutants and the lung cancer incidence and mortality rates, SO_2_ and NO_2_ were associated with MAIR, MAMR and FAMR. For PM_10_, there was no significant correlation with the male lung cancer incidence and mortality rates. NO_2_ presented higher RRs than SO_2_ for MAIR, MAMR and FAMR, suggesting stronger impact of NO_2_ on the burden of lung cancer.

There were reports of the correlation between NO_2_ exposure and cancer incidence rates in Europe and America (48, 49). NO_2_ has a significant positive correlation with cancer incidence rates, especially lung cancer, prostate cancer and breast cancer (49, 50). In a Danish study, it was demonstrated that NO_2_ concentration was more associated with lung squamous cell carcinoma and small cell carcinoma than lung adenocarcinoma (51). A population-based case-control study including 908 lung cancer patients and 908 controls provided positive evidence for the association between exposure to ambient air pollution and lung cancer incidence in Koreans. The increase in the lung cancer incidence (OR) was 1.10 (95% CI: 1.00-1.22) for every 10 μg/m^3^ increase in NO_2_. Stronger associations between air pollution and lung cancer incidence were noted among never smokers and those with low fruit consumption (50).

Compared with that in developed countries in Europe and the United States, the air pollution in China is undoubtedly high. This study covers a wide range of regions and population in China, providing important information that NO_2_ is closely related to the incidence and mortality of lung cancer. It is warranted that more attention be paid to promote national policies and raise public awareness of pollutant control, especially of NO_2_.

There are only a few studies on the correlation between SO_2_ and the tumor incidence rate and results of most studies were negative (52). Our study found that SO_2_ is closely related to MAIR, MAMR and FAMR of lung cancer. This outcome suggests that China should pay attention to the control of SO_2_ and to other air pollutants, and thus indirectly control the incidence rate and mortality of lung cancer.

Our study found that there was no strong evidence associating PM_10_ with the incidence rate and mortality of lung cancer. Some studies from low-pollution areas in Europe and America have investigated the relationship between PM_10_ and cancer incidence rate, most of which focused on lung cancer (49, 53, 54). A few studies evaluated the effects of PM_10_ on nonlung cancer mortality, including breast cancer (55), nasopharyngeal carcinoma (56), liver cancer, colorectal cancer, bladder cancer, and kidney cancer mortality (57). Hamra et al. provided meta-analysis of increased lung cancer risk associated with exposure to PM in outdoor air from 17 cohort studies (14). An increase of 7 μg/m^3^ in PM_10_ was associated with an increased HR of 1.84 for lung cancer mortality (95% CI: 1.23-2.74) (49). However, not all studies are consistent. In their case-control studies of Europeans, Vineis et al. found a nonsignificant correlation between PM_10_ and lung cancer incidence rate (52).

Because of the imperfection of air pollution detection systems in China, the detection of PM_2.5_ started late. It was not available to obtain the relatively complete data of PM_2.5_ in our study. Whether PM_2.5_ is closely related to the incidence and mortality of lung cancer remains to be further studied. Our study showed that the chemical components of air pollutants, such as NO_2_ and SO_2_, seem to contribute more to the burden of lung cancer than PM_10_. Therefore, the control of exhaust emissions is of parallel importance with the prevention of haze particles.

Our results showed that PM_10_ had no significant correlation with the lung cancer MAIR and FAIR, while the YAV of SO_2_ and NO_2_ 2 years before the outcome and the YAV of NO_2_ during the present year were significant risk factors for the lung cancer MAIR, especially the YAV of SO_2_ and NO_2_ 2 years before the outcome, suggesting that NO_2_ and SO_2_ may have a 2-year lag in their effects on male lung cancer. The cumulative time effect of environmental pollutants on tumor incidence rate presents an inverted U structure. With the exposure period getting longer, the impact of environmental pollutants on the tumor incidence rate increased in the first period and reached the highest level. After the peak, it decreased gradually. The strongest effect points of PM_10_, SO_2_ or NO_2_ on lung cancer MAIR are at the time of 2 years before the cancer occurrence. The most important factor affecting the incidence rate of MAIR is industrial NO_2_.

In addition to cancer incidence, lung cancer mortality is another end point in the study. There is insufficient evidence of whether ambient air pollution may be related to cancer progression or survival. One recent study in California enrolled >350,000 lung cancer patients and reported that higher residential ambient air pollution concentrations (NO_2_, PM_2.5_, PM_10_) were associated with poorer survival, particularly among patients diagnosed in earlier disease states (i.e., with localized disease) (2).

Lung cancer is rapidly fatal with 5-year survival rates of 18%, thus, the use of mortality data reasonably approximates disease incidence. Actually, survival is of greater significance for lung cancer, which reflects both disease incidence and survival following diagnosis. Our results suggest that NO_2_ and SO_2_ levels three years before cancer occurrence are most closely related to the lung cancer MAMR and FAMR. PM_10_ had no significant effect on the mortality of lung cancer in males, but had a certain correlation with the mortality of females. The cumulative time effect of environmental pollutants on the lung cancer mortality rate also presents an inverted-U structure. With exposure period getting longer, the impact of environmental pollutants on tumor incidence rate increased in the first period and reached the highest level. After the peak, it decreased gradually. The strongest effect points of SO_2_ and NO_2_ on lung cancer MAMR and FAMR are 3 years before the cancer occurrence. The data in **Figure 3** show that the most important factor affecting the incidence rate of MAMR and FAMR is industrial NO_2_.

The study has the following limitations: (1) China enforces a household register system and the annual data of China cancer registration are collected from permanent resident population in each city, lacking data from the transient population in the city. These seven cities are important representative cities in China with large transient populations and the transient population was more likely to participate in outdoor activities. Thus, a part of the population of each city was not included in the analysis, discounting the accuracy of the results. (2) The mechanism of lung cancer is not fully understood. The risk factors are diverse and complex and there are inherent differences in population susceptibility. The mortality of lung cancer is also affected by many aspects including timely diagnosis and active treatment. Although air pollutants have adverse effects on public health, the analysis of their correlation with lung cancer may be interfered by biological (e.g., physiopathological responses), demographic and behavioral differences (e.g., type of occupation, smoking, and lifestyle) (5). Although some of the confounding factors can be eliminated by horizontal comparison in multiple cities, this study was not be stratified by other potential confounding factors (such as smoking, lifestyle, obesity and socioeconomic status) other than age and gender. Therefore, to some degree the conclusions obtained are one-sided. (3) Although the incidence of lung cancer in rural areas is lower than that in urban areas, the former is on the rise. The main components of air pollution in rural areas may be different from those in cities. As China has a vast territory with a large population, at present, air pollution detection points have been only established in cities and suburbs and do not cover rural areas. Thus, this study did not involve data from rural areas and the relationship between air pollutants and lung cancer occurrence and mortality in rural areas needs further study.

With the rapid development of China’s economy, air pollution has become a threatening public health problem (19). Many experiments and epidemiological studies have shown that air pollution has health hazards, such as carcinogenesis, cardiovascular and respiratory harms. It is an urgent and important task to evaluate the correlation between air pollution and disease-specific incidence and mortality rates in China. Our research demonstrated that the lung cancer incidence and mortality rates were consistent with the degree of air pollution, based on air pollutant data and lung cancer registration data from seven cities in China. PM_10_, SO_2_ and NO_2_ were selected as air pollution monitoring indicators. Among them, NO_2_ was the most closely related to lung cancer, followed by SO_2_, and PM_10_ exhibited the weakest effects. Air pollutants have the strongest cumulative effect on the incidence and mortality of lung cancer at 2-3 years of exposure. There are gender differences in air pollution-associated lung cancer risks. The air pollutant presented stronger effects on males than females and males may be more sensitive to air pollutants than females.

Air pollutants have very complex physical and chemical properties and have strong temporal and spatial heterogeneity. There are far more components of air pollution than PM_10_, SO_2_ and NO_2_. To formulate corresponding environmental protection measures, it is necessary to identify the components of air pollution and to explore the major sources of air pollution emission. Further research needs to be conducted to evaluate the health hazards of individual components, aiming at providing guidance for environmental pollution control and public health protection. The association between lung cancer burden and air pollution may shift public perceptions and ultimately help to promote policy development on air quality.

Besides air pollution, there is a lot of risk factor for lung cancer, such as smoking (58), family history of lung cancer, and so on, which were the confounders. More than 50% of men smoke in China, and there are more than 300 million smokers in China (59). Smoking and air pollution combined to account for the elevated rates of lung cancer mortality in Shenyang of China (60). Family history of lung cancer, history of tuberculosis are also the independent risk factors for lung cancer (61). These risk factors are confounders, but in this study, we focused on the air pollutants and performed univariate analysis without considering other factors. Because the air pollutants are too complex to perform multivariate analysis.

## Conclusions

Our research demonstrated that the lung cancer incidence and mortality rates were consistent with the degree of air pollution, based on air pollutant data and lung cancer registration data from seven cities in China. NO_2_ was the most closely related to lung cancer, followed by SO_2_ and then PM_10_. Air pollutants have the strongest cumulative effect on the incidence and mortality of lung cancer at 2-3 years of exposure.

## Data availability statement

The original contributions presented in the study are included in the article/**Supplementary Material**. Further inquiries can be directed to the corresponding author.

## Ethics statement

This study was approved by the Ethics Committee of Hunan Cancer Hospital. The lung cancer incidence and mortality data were extracted from the “China Cancer Registry Annual Report, 2009~2017” published by the National Cancer Registry Center, research and academic use permitted.

## Author contributions

WW provided the idea and designed the article. LM and ZH collected the data. WW, LM, ZH, XY, WZ, KL, HL and MT analyzed the data. XZ, XT, CL, YH and SY edited the figures. WW, LM and XY wrote the manuscript. All authors contributed to the article and approved the submitted version.

## Funding

The Clinical Research Center For Gastrointestinal Cancer In Hunan Province [No.2021SK4016], Project of Hunan Provincial Health Commission [No.202203105045, No. C2014-34 and No. 20201665], Hunan Provincial Natural Science Foundation of China [No.2022JJ40257], Project of Chinese Society of Clinical Oncology (Y-HR2018-234), Project of Hunan Cancer Hospital (No. A2011-03), and “Scientific Research Climbing Plan” of Hunan Cancer Hospital [No. 2020NSFC-B005].

## Conflict of interest

The authors declare that the research was conducted in the absence of any commercial or financial relationships that could be construed as a potential conflict of interest.

## Publisher’s note

All claims expressed in this article are solely those of the authors and do not necessarily represent those of their affiliated organizations, or those of the publisher, the editors and the reviewers. Any product that may be evaluated in this article, or claim that may be made by its manufacturer, is not guaranteed or endorsed by the publisher.

## Abbreviations

PM, particulate matter; SO_2_, sulfur dioxide; NO_2_, nitrogen dioxide; CSYB, China Statistical Yearbook; NDE, National Department of Environment; YAV, yearly average values; CCRAR, Chinese Cancer Registration Annual Report; CIR, crude incidence rate; CMR, crude mortality rate; MAIR, male age-adjusted incidence rate; FAIR, female age-adjusted incidence rate; MAMR; male age-adjusted mortality rate; FAMR, female age-adjusted mortality rate.
