# Supplementary material for: The association between outdoor air pollution and lung cancer risk in seven eastern metropolises of China: Trends in 2006-2014 and sex differences
Source: Front Oncol. 2022 Sep 29;12:939564. doi: 10.3389/fonc.2022.939564 (PMC9556871; doi:10.3389/fonc.2022.939564)
Supplement: Supplementary file 1 [file Table_1.docx]

| **Supplementary Table 1. The association between the yearly concentrations of PM_10_, SO_2_, NO_2_ within a 5-year moving window and CIR, CMR of lung cancer.** | | | |
| --- | --- | --- | --- |
| **Statistics** | **Covariates** | **RR (95% CI)** | **p-value** |
| **CIR** | PM_10_ 5 years before | 0.88 (0.69, 1.13) | 0.34 |
|  | SO_2_ 5 years before | 0.80 (0.53, 1.27) | 0.31 |
|  | NO_2_ 5 years before | 0.76 (0.45, 1.27) | 0.31 |
|  | PM_10_ 4 years before | 0.79 (0.61, 1.01) | 0.07 |
|  | SO_2_ 4 years before | 0.65 (0.48, 0.88) | 0.01 |
|  | NO_2_ 4 years before | 0.65 (0.40, 1.07) | 0.10 |
|  | PM_10_ 3 years before | 0.78 (0.63, 0.97) | 0.03 |
|  | SO_2_ 3 years before | 0.79 (0.60, 1.04) | 0.10 |
|  | NO_2_ 3 years before | 1.16 (0.72, 1.85) | 0.54 |
|  | PM_10_ 2 years before | 0.80 (0.66, 0.97) | 0.03 |
|  | SO_2_ 2 years before | 0.78 (0.62, 0.99) | 0.04 |
|  | NO_2_ 2 years before | 0.74 (0.48, 1.13) | 0.18 |
|  | PM_10_ 1 year before | 0.85 (0.70, 1.02) | 0.09 |
|  | SO_2_ 1 year before | 0.78 (0.64, 0.95) | 0.02 |
|  | NO_2_ 1 year before | 0.58 (0.38, 0.88) | 0.01 |
|  | PM_10_ at the present year | 0.84 (0.69, 1.01) | 0.07 |
|  | SO_2_ at the present year | 0.76 (0.64, 0.90) | 0.002 |
|  | NO_2_ at the present year | 0.67 (0.44, 1.01) | 0.06 |
| **CMR** | PM_10_ 5 years before | 0.96 (0.83, 1.12) | 0.63 |
|  | SO_2_ 5 years before | 0.98 (0.80, 1.20) | 0.84 |
|  | NO_2_ 5 years before | 0.96 (0.73, 1.27) | 0.79 |
|  | PM_10_ 4 years before | 0.95 (0.84, 1.08) | 0.48 |
|  | SO_2_ 4 years before | 0.92 (0.80, 1.05) | 0.24 |
|  | NO_2_ 4 years before | 1.04 (0.82, 1.32) | 0.75 |
|  | PM_10_ 3 years before | 0.91 (0.78, 1.06) | 0.24 |
|  | SO_2_ 3 years before | 1.01 (0.84, 1.21) | 0.93 |
|  | NO_2_ 3 years before | 1.23 (0.90, 1.68) | 0.21 |
|  | PM_10_ 2 years before | 0.85 (0.73, 0.99) | 0.04 |
|  | SO_2_ 2 years before | 0.88 (0.73, 1.05) | 0.16 |
|  | NO_2_ 2 years before | 0.62 (0.45, 0.85) | 0.01 |
|  | PM_10_ 1 year before | 0.87 (0.76, 1.01) | 0.07 |
|  | SO_2_ 1 year before | 0.83 (0.72, 0.96) | 0.02 |
|  | NO_2_ 1 year before | 0.52 (0.38, 0.70) | <0.0001 |
|  | PM_10_ at the present year | 0.86 (0.75, 1.00) | 0.06 |
|  | SO_2_ at the present year | 0.82 (0.72, 0.94) | 0.004 |
|  | NO_2_ at the present year | 0.84 (0.60, 1.17) | 0.30 |
| Note: RR represents rate ratio: the ratio of the incidence and mortality rate at one-unit increase of numeric variable versus the incidence and mortality rate at baseline. RR larger than 1 suggested a risk effect, while RR less than 1 suggested a protective effect. P value was calculated by using the two-level random intercept regression analysis. | | | |
