# Supplementary material for: The association between outdoor air pollution and lung cancer risk in seven eastern metropolises of China: Trends in 2006-2014 and sex differences
Source: Front Oncol. 2022 Sep 29;12:939564. doi: 10.3389/fonc.2022.939564 (PMC9556871; doi:10.3389/fonc.2022.939564)
Supplement: Supplementary file 2 [file Table_2.docx]

| **Supplementary Table 2. The association between the yearly concentrations of PM_10_, SO_2_, NO_2_ within a 5-year moving window and FAIR, FAMR of lung cancer.** | | | |
| --- | --- | --- | --- |
| **Statistics** | **Covariates** | **RR (95% CI)** | **p-value** |
| **FAIR** | PM_10_ 5 years before | 0.97 (0.89, 1.05) | 0.40 |
|  | SO_2_ 5 years before | 0.88 (0.78, 0.99) | 0.05 |
|  | NO_2_ 5 years before | 0.91 (0.78, 1.08) | 0.30 |
|  | PM_10_ 4 years before | 0.96 (0.89, 1.04) | 0.30 |
|  | SO_2_ 4 years before | 0.93 (0.83, 1.03) | 0.16 |
|  | NO_2_ 4 years before | 0.97 (0.82, 1.14) | 0.70 |
|  | PM_10_ 3 years before | 0.99 (0.92, 1.07) | 0.80 |
|  | SO_2_ 3 years before | 0.95 (0.86, 1.05) | 0.29 |
|  | NO_2_ 3 years before | 0.96 (0.81, 1.13) | 0.60 |
|  | PM_10_ 2 years before | 1.01 (0.94, 1.09) | 0.71 |
|  | SO_2_ 2 years before | 0.97 (0.89, 1.07) | 0.58 |
|  | NO_2_ 2 years before | 1.01 (0.85, 1.19) | 0.91 |
|  | PM_10_ 1 year before | 1.03 (0.96, 1.10) | 0.46 |
|  | SO_2_ 1 year before | 0.96 (0.89, 1.04) | 0.34 |
|  | NO_2_ 1 year before | 0.98 (0.83, 1.16) | 0.79 |
|  | PM_10_ at the present year | 1.00 (0.93, 1.07) | 0.96 |
|  | SO_2_ at the present year | 0.97 (0.90, 1.03) | 0.29 |
|  | NO_2_ at the present year | 0.97 (0.83, 1.13) | 0.69 |
| **FAMR** | PM_10_ 5 years before | 1.03 (0.95, 1.13) | 0.46 |
|  | SO_2_ 5 years before | 1.08 (0.97, 1.21) | 0.19 |
|  | NO_2_ 5 years before | 1.16 (1.00, 1.34) | 0.05 |
|  | PM_10_ 4 years before | 1.07 (0.99, 1.17) | 0.09 |
|  | SO_2_ 4 years before | 1.09 (0.99, 1.19) | 0.09 |
|  | NO_2_ 4 years before | 1.20 (1.04, 1.39) | 0.02 |
|  | PM_10_ 3 years before | 1.09 (1.01, 1.19) | 0.04 |
|  | SO_2_ 3 years before | 1.13 (1.03, 1.24) | 0.02 |
|  | NO_2_ 3 years before | 1.27 (1.08, 1.49) | 0.01 |
|  | PM_10_ 2 years before | 1.09 (1.02, 1.17) | 0.02 |
|  | SO_2_ 2 years before | 1.08 (0.99, 1.17) | 0.07 |
|  | NO_2_ 2 years before | 1.06 (0.90, 1.24) | 0.52 |
|  | PM_10_ 1 year before | 1.06 (0.99, 1.13) | 0.11 |
|  | SO_2_ 1 year before | 1.03 (0.96, 1.11) | 0.43 |
|  | NO_2_ 1 year before | 0.97 (0.83, 1.15) | 0.75 |
|  | PM_10_ at the present year | 1.01 (0.95, 1.08) | 0.71 |
|  | SO_2_ at the present year | 1.02 (0.96, 1.08) | 0.59 |
|  | NO_2_ at the present year | 1.05 (0.91, 1.22) | 0.48 |
| Note: RR represents rate ratio: the ratio of the incidence and mortality rate at one-unit increase of numeric variable versus the incidence and mortality rate at baseline. RR larger than 1 suggested a risk effect, while RR less than 1 suggested a protective effect. P value was calculated by using the two-level random intercept regression analysis. | | | |
|  |  |  |  |
