# Supplementary material for: The association between outdoor air pollution and lung cancer risk in seven eastern metropolises of China: Trends in 2006-2014 and sex differences
Source: Front Oncol. 2022 Sep 29;12:939564. doi: 10.3389/fonc.2022.939564 (PMC9556871; doi:10.3389/fonc.2022.939564)
Supplement: Supplementary file 3 [file Table_3.docx]

| **Supplementary Table 3. The association between the yearly concentrations of PM_10_, SO_2_, NO_2_ within a 5-year moving window and MAIR, MAMR of lung cancer.** | | | |
| --- | --- | --- | --- |
| **Statistics** | **Covariates** | **RR (95% CI)** | **p-value** |
| **MAIR** | PM_10_ 5 years before | 0.97 (0.81, 1.15) | 0.71 |
|  | SO_2_ 5 years before | 0.91 (0.70, 1.19) | 0.50 |
|  | NO_2_ 5 years before | 0.79 (0.56, 1.11) | 0.19 |
|  | PM_10_ 4 years before | 0.92 (0.78, 1.07) | 0.29 |
|  | SO_2_ 4 years before | 1.07 (0.88, 1.30) | 0.50 |
|  | NO_2_ 4 years before | 1.32 (0.97, 1.79) | 0.08 |
|  | PM_10_ 3 years before | 1.00 (0.86, 1.17) | 0.996 |
|  | SO_2_ 3 years before | 1.10 (0.92, 1.32) | 0.31 |
|  | NO_2_ 3 years before | 1.28 (0.94, 1.74) | 0.13 |
|  | PM_10_ 2 years before | 1.07 (0.94, 1.23) | 0.32 |
|  | SO_2_ 2 years before | 1.20 (1.03, 1.39) | 0.02 |
|  | NO_2_ 2 years before | 1.57 (1.19, 2.05) | 0.002 |
|  | PM_10_ 1 year before | 1.07 (0.93, 1.22) | 0.36 |
|  | SO_2_ 1 year before | 1.11 (0.96, 1.28) | 0.16 |
|  | NO_2_ 1 year before | 1.18 (0.86, 1.62) | 0.32 |
|  | PM_10_ at the present year | 1.07 (0.94, 1.23) | 0.30 |
|  | SO_2_ at the present year | 1.10 (0.98, 1.24) | 0.12 |
|  | NO_2_ at the present year | 1.38 (1.04, 1.83) | 0.03 |
| **MAMR** | PM_10_ 5 years before | 1.02 (0.90, 1.15) | 0.76 |
|  | SO_2_ 5 years before | 1.07 (0.91, 1.25) | 0.44 |
|  | NO_2_ 5 years before | 1.20 (0.97, 1.47) | 0.10 |
|  | PM_10_ 4 years before | 1.05 (0.92, 1.20) | 0.50 |
|  | SO_2_ 4 years before | 1.10 (0.95, 1.28) | 0.22 |
|  | NO_2_ 4 years before | 1.30 (1.03, 1.64) | 0.04 |
|  | PM_10_ 3 years before | 1.09 (0.94, 1.26) | 0.24 |
|  | SO_2_ 3 years before | 1.27 (1.09, 1.49) | 0.004 |
|  | NO_2_ 3 years before | 1.70 (1.32, 2.18) | 0.0002 |
|  | PM_10_ 2 years before | 1.09 (0.96, 1.24) | 0.18 |
|  | SO_2_ 2 years before | 1.20 (1.04, 1.38) | 0.02 |
|  | NO_2_ 2 years before | 1.24 (0.94, 1.63) | 0.14 |
|  | PM_10_ 1 year before | 1.06 (0.93, 1.20) | 0.38 |
|  | SO_2_ 1 year before | 1.08 (0.95, 1.24) | 0.23 |
|  | NO_2_ 1 year before | 0.96 (0.71, 1.29) | 0.77 |
|  | PM_10_ at the present year | 1.05 (0.93, 1.18) | 0.43 |
|  | SO_2_ at the present year | 1.07 (0.96, 1.19) | 0.24 |
|  | NO_2_ at the present year | 1.27 (0.99, 1.64) | 0.06 |
| Note: RR represents rate ratio: the ratio of the incidence and mortality rate at one-unit increase of numeric variable versus the incidence and mortality rate at baseline. RR larger than 1 suggested a risk effect, while RR less than 1 suggested a protective effect. P value was calculated by using the two-level random intercept regression analysis. | | | |
|  |  |  |  |
